# Structure specific recognition of telomeric repeats containing RNA by the RGG-box of hnRNPA1

**DOI:** 10.1093/nar/gkaa134

**Published:** 2020-03-04

**Authors:** Meenakshi Ghosh, Mahavir Singh

**Affiliations:** 1 Molecular Biophysics Unit, Indian Institute of Science, Bengaluru, 560012, India; 2 NMR Research Centre, Indian Institute of Science, Bengaluru, 560012, India

## Abstract

The telomere repeats containing RNA (TERRA) is transcribed from the C-rich strand of telomere DNA and comprises of UUAGGG nucleotides repeats in humans. The TERRA RNA repeats can exist in single stranded, RNA-DNA hybrid and G-quadruplex forms in the cell. Interaction of TERRA RNA with hnRNPA1 has been proposed to play critical roles in maintenance of telomere DNA. hnRNPA1 contains an N-terminal UP1 domain followed by an RGG-box containing C-terminal region. RGG-motifs are emerging as key protein motifs that recognize the higher order nucleic acid structures as well as are known to promote liquid-liquid phase separation of proteins. In this study, we have shown that the RGG-box of hnRNPA1 specifically recognizes the TERRA RNA G-quadruplexes that have loops in their topology, whereas it does not interact with the single-stranded RNA. Our results show that the N-terminal UP1 domain in the presence of the RGG-box destabilizes the loop containing TERRA RNA G-quadruplex efficiently compared to the RNA G-quadruplex that lacks loops, suggesting that unfolding of G-quadruplex structures by UP1 is structure dependent. Furthermore, we have compared the telomere DNA and TERRA RNA G-quadruplex binding by the RGG-box of hnRNPA1 and discussed its implications in telomere DNA maintenance.

## INTRODUCTION

The C-rich strand of telomere DNA in eukaryotes is transcribed into a long non-coding RNA called the telomeric repeats containing RNA (TERRA) (1–3). Due to the repeated nature of the telomere DNA (TTAGGG deoxynucleotides repeats in vertebrates), the TERRA RNA sequence consists of UUAGGG nucleotide repeats. TERRA RNA has been shown to localize at the telomere ends and play essential roles in telomere maintenance, heterochromatin formation, and replication (4,5). TERRA RNA repeats have been found to bind a number of proteins that include telomerase reverse transcriptase (hTERT), telomeric repeat binding factor-2 (TRF2) and heterogenous nuclear ribonucleoprotein A1 (hnRNPA1) (6,7). Complexity of TERRA functions also stems from the observation that TERRA repeats can exist in single-stranded, RNA-DNA hybrid, and RNA G-quadruplex forms in the cell (8). The G-quadruplex forms can further exist in structures of different topologies with intramolecular G-quadruplex being the most physiologically relevant form. Therefore, how a TERRA binding protein recognizes the TERRA sequences in different conformations is an important question.

**Figure 1.**
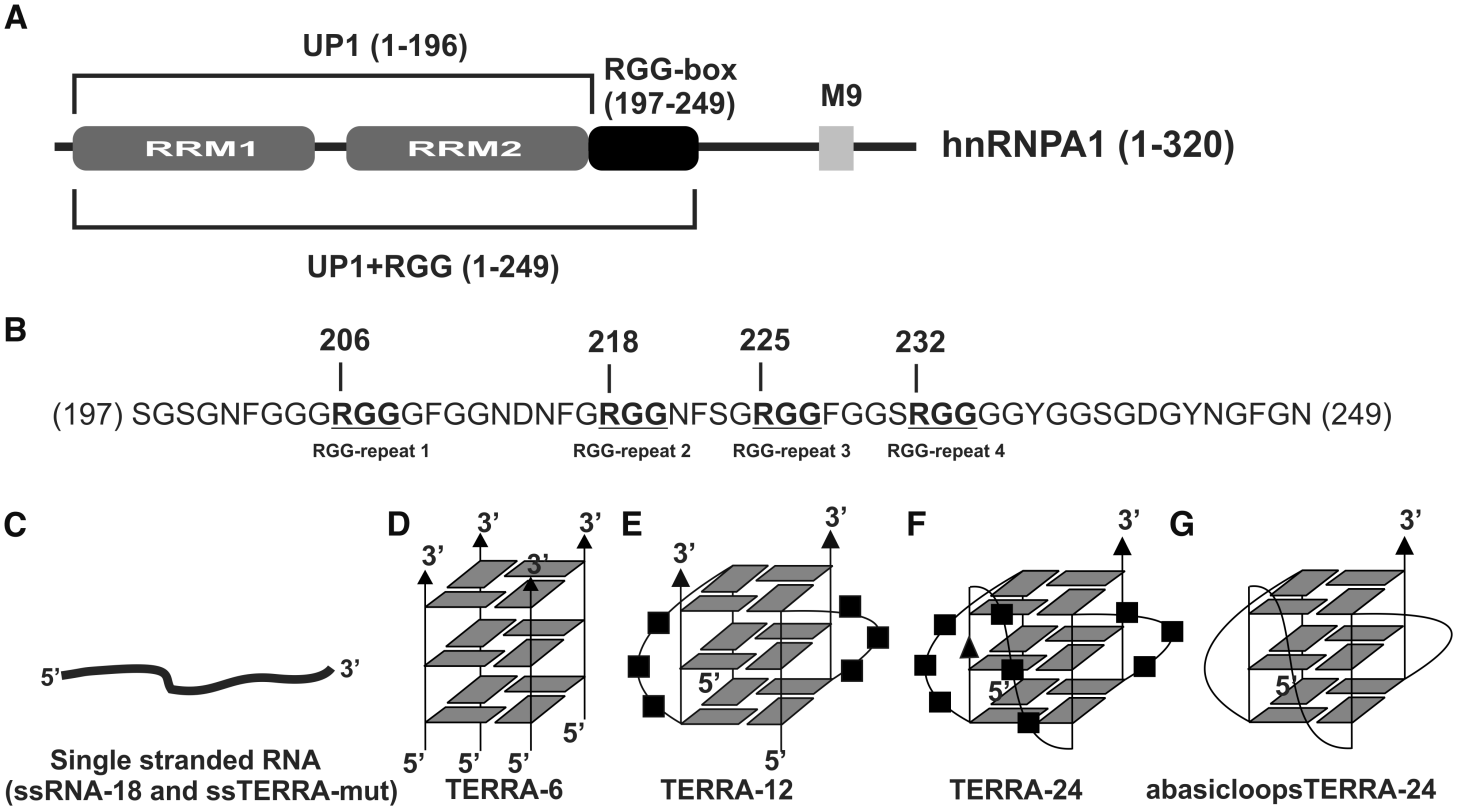
Cartoon representation of hnRNPA1 and RNA sequences used in this study. (**A**) Domain architecture of hnRNPA1 depicting different domains used in this study. (**B**) Primary sequence of the RGG-box of hnRNPA1. The four RGG repeats present in hnRNPA1 RGG-box are maked in bold and underlined. (C–G) Cartoon representation of different RNA sequences used for the study. ssTERRA-mut and ssRNA-18 do not form any secondary structure in the presence of 100 mM KCl and remain in single-stranded conformation (**C**). TERRA-6 (**D**) forms parallel tetrameric RNA G-quadruplex whereas TERRA-12 (**E**) and TERRA-24 (**F**) form dimeric and intramolecular parallel G-quadruplexes respectively in the presence of 100 mM KCl. AbasicloopsTERRA-24 (**G**) also forms a parallel intramolecular G-quadruplex structure which lacks bases in its loop.

hnRNPA1 is an abundant protein in the eukaryotic cell that is involved in telomere DNA maintenance apart from its roles in RNA splicing, stability and transport (9–11). The N terminus of hnRNPA1 consists of two RNA binding domains, namely RRM1 and RRM2, collectively called the UP1 domain. UP1 is followed by a C-terminal region that contains an RGG-box, a prion-like domain, and a M9 nuclear shuttling sequence (Figure 1A). In RGG-box containing proteins, typically two RGG repeats are separated by 0–4 intervening amino acid residues, though there are also reports of RGG repeats being separated by up to nine residues (12–14). In hnRNPA1, the RGG-box consists of four RGG repeats comprising of a tri-RGG motif (RGG-X_4_-RGG-X_4_-RGG, where X is any amino acid) and a single RGG repeat that is present nine amino acids away towards the N terminal region of the tri-RGG motif (Figure 1B).

The UP1 domain has been shown to interact with both telomere DNA and TERRA RNA repeats and regulate the telomerase activity. These interactions have been proposed to have multiple roles pertaining to telomere DNA protection (15–21). Since TERRA RNA repeats are complementary to the template sequence of the telomerase RNA (hTR), TERRA has also been shown to directly inhibit the telomere repeat elongation activity of telomerase holoenzyme by binding to the template region of the hTR. Consequently, a three-state model for the regulation of telomerase activity was proposed, where hnRNPA1 was propounded to alleviate the TERRA mediated telomerase inhibition, by directly binding to and titrating away the TERRA RNA (22). Single-strand DNA binding protein RPA (Replication Protein A), which is a key sensor of DNA damage during DNA repair and recombination (23–25) is replaced by Protection of Telomere-1 (POT1) protein at single stranded telomere DNA. This is essential for telomere DNA end protection and genomic stability (25,26). TERRA and hnRNPA1 interaction has also been implicated in controlling the RPA to POT1 switch at the telomere ends (27).

In recent studies, hnRNPA1 was shown to interact with the TERRA RNA repeats (28,29). However, a quantitative characterization of this interaction as well as the contribution of the individual UP1 domain and the RGG-box in TERRA RNA recognition remains missing. The recognition of different TERRA RNA sequences (single stranded and the various forms of G-quadruplexes) by UP1 and RGG-box has not been defined in energetic terms. UP1 has been established as a telomere DNA G-quadruplex binding and destabilizing protein (17,21,30). However, the unfolding of TERRA RNA G-quadruplex by UP1 and UP1+RGG has not been probed systematically.

RGG-boxes are emerging as key higher order DNA/RNA structure binding motifs (31). In several proteins, the RGG-boxes are reported to enhance the affinity as well as the specificity of RNA-protein interaction (32–34). The binding of RGG-box to the G-quadruplex has been shown to destabilize G-quadruplex structure in some cases, while in other cases it has been shown to stabilize the G-quadruplex structure. For example, the RGG-box of FMRP has been reported to stabilize the higher order RNA structures formed by sc1 RNA (35,36) whereas it unfolds the RNA G-quadruplex formed by S3F RNA (37). Very recently, a study showed that the association between the RGG-box of FMRP and the sc1 RNA G-quadruplex is enhanced in the presence of RNA helicase Moloney leukemia virus 10 (MOV10), suggesting that other trans factors may be involved in modulating these interactions in vivo (38). Telomere DNA binding FUS/TLS is an oncogenic human protein that contains three RGG-boxes in its sequence. Interestingly, these three RGG-domains have been shown to have distinct nucleic acid binding properties (39). While RGG1 and RGG2 were shown to not bind the telomere DNA G-quadruplex, the RGG3 of TLS was shown to bind and stabilize both the telomere DNA and TERRA RNA G-quadruplex structures (39). Furthermore, a recent study showed that the RGG2 of FUS/TLS binds and destabilizes the 5′ end of the loop of a RNA stem loop structure (33). The RGG-box containing C-terminal domain of nucleolin was shown to bind the c-myc DNA G-quadruplex with a high affinity thereby stabilizing the G-quadruplex formation (40). hnRNPA1 was shown to bind and unfold the G-quadruplex from the human KRAS promoter and telomere DNA (17,41). Recently, we had shown that the RGG-box of hnRNPA1 binds specifically to the telomere DNA G-quadruplex and enhances the DNA G-quadruplex unfolding by UP1 (30). All these studies showed the complex nature of RGG-box interactions with DNA/RNA G-quadruplexes resulting in specific outcome in different proteins.

Here, we have probed the role of the RGG-box of hnRNPA1 in TERRA RNA recognition. We have shown that the RGG-box of hnRNPA1 binds to the TERRA RNA G-quadruplexes in a structure dependent manner. Using NMR spectroscopy, we have elucidated that the RGG-box of hnRNPA1 primarily binds to the loop containing intramolecular RNA G-quadruplex formed by the TERRA RNA repeats, while it does not interact with the single-stranded RNA sequences. The binding of the RGG-box with TERRA RNA G-quadruplex is ∼31-fold stronger than its binding to the telomere DNA G-quadruplex. Furthermore, we have shown that the hnRNPA1 destabilizes the TERRA RNA G-quadruplexes in a structure dependent manner where the RGG-box enhances the G-quadruplex unfolding efficiency of the UP1 domain.

## MATERIALS AND METHODS

### Protein purification and preparation of RNA samples

The DNA sequences encoding UP1 (residues 1–196) and UP1+RGG (residues 1–249) of hnRNPA1 (Uniprot identifier P09651-2) were sub cloned into pET28a E. coli expression vector. Both the constructs contain a C-terminal hexa-histidine (6x His) affinity purification tag. The proteins were expressed in BL21 Rosetta (DE3) cells in LB media. The cells were induced with 0.5 mM IPTG and incubated for overnight at 30°C after growing them at 37°C till the culture reached an optical density (OD) at 600 nm of 0.6. First step of purification involved 6x His-tag–Ni-NTA affinity chromatography followed by cation exchange and size exclusion chromatography (SEC) using a Superdex 75 column. The isolated RGG-box was sub cloned into pGEX-6P-1 vector and a TEV cleavage site was introduced between the GST tag and the protein. Glutathione-sepharose column was used for the purification of RGG-box, followed by the GST tag removal using TEV protease. SEC using Superdex 75 column was performed at the final step of purification of the RGG-box. The final purity of all the proteins was ascertained using SDS-PAGE and MALDI-MS analyses and the proteins were adjudged to be >95% purified.

The RNA sequences were ordered from Eurofins (Supplementary Table S1). For ITC and CD experiments the RNA samples were prepared in 20 mM Tris, pH 8.0 and 100 mM KCl. For the NMR titration experiments 10 mM potassium phosphate (pH 6.5) and 100 mM KCl was used as the buffer composition to prepare the RNA samples. The RNA samples were heated at 95°C for 3 min and then cooled at 4°C for overnight. The concentration of the RNA samples was calculated by ultraviolet (UV) absorbance at 260 nm using Eppendorf Spectrophotometer. The formation of the G-quadruplex by 6mer TERRA-6, 12mer TERRA-12, 24mer TERRA-24 RNA and 24mer abasicloopsTERRA-24 was confirmed by recording spectral scan and thermal melting using CD spectroscopy as well as by the presence of the imino proton peaks in the 1D ^1^H NMR spectra.

### Isothermal titration calorimetry

Isothermal titration calorimetry (ITC) experiments were performed using iTC200 instrument (GE) at 25°C. The protein and the RNA samples were thoroughly de-gassed before the experiment. For the titrations, 100–200 μM of the protein was titrated into 5 μM of the RNA filled in the sample cell. 20 injections containing 2 μl of the titrant each were made keeping an interval of 180 s between each injection. The integrated heat data for all the runs was corrected for the heat of dilution, the protein being injected at each step. The data was then fitted for one site binding model using ORIGIN software provided by the vendor (GE). All the parameters were kept floating during the data fitting. All the experiments were repeated at least two times for data consistency.

### Circular dichroism spectroscopy

The structural changes in RNA G-quadruplexes (TERRA-6, TERRA-12, TERRA-24, abasicloopsTERRA-24) by UP1 and UP1+RGG were monitored using CD spectroscopy. CD titrations were carried out using JASCO J-715 Spectrophotometer. The wavelength scans for the RNAs were done from 325 to 225 nm. The average of three spectral scans was taken for each titration step. Baseline correction was done for each run and the data was normalized to molar ellipticity per residue. The titration experiments were performed in 1 cm path length cuvette from Hellma Analytics at 25°C at a scanning speed of 100 nm/min with a response time of 4 s. 5 μM of the RNA sample was titrated with increasing protein concentration (in the steps of 0, 2.5 μM, 5 μM, 10 μM, 15 μM, 20 μM and 25 μM of proteins).

For the melting studies of the RNA G-quadruplexes, the change in ellipticity was measured at 262 nm as a function of increasing temperature. The ellipticity was monitored from 10°C to 100°C with an increase of temperature of 1°C every minute. Data points were recorded after every 0.5°C.

### NMR spectroscopy

1D ^1^H NMR and 2D ^1^H–^15^N HSQC spectra of protein and 1D ^1^H NMR spectra of RNA were recorded on Bruker 700 MHz, equipped with a cryoprobe, at 298 K. Water suppression for the 1D ^1^H experiments was done using excitation-sculpting. 550 μl of 50 μM of RNA sample was prepared in 10 mM potassium phosphate (pH 6.5) containing 100 mM KCl. The RNA was then titrated with increasing concentration of UP1 and UP1+RGG (in K^+^ buffer).

For the 2D ^1^H–^15^N HSQC titration experiments, 500 μl of 138 μM of the ^15^N labeled RGG-box sample was prepared in 10 mM potassium phosphate (pH 6.5) and 100 mM KCl. 10% D_2_O was added to the sample for the deuterium lock. The protein was titrated with increasing concentration of RNA (TERRA-24, TERRA-12, TERRA-6, ssTERRA-mut and ssRNA-18) followed by 10 min of incubation. In case of abasicloopsTERRA-24 titration with the RGG-box, 120 μM of ^15^N labeled RGG-box sample was titrated with increasing concentration of the RNA G-quadruplex. 2D ^1^H–^15^N HSQC spectrum of the protein was recorded at each step of the titration. The chemical shift changes were monitored in each step of the titration. The NMR data was processed using Bruker TOPSPIN 3.1 and analyzed using NMRFAM-SPARKY (42).

### Analysis of NMR chemical shift perturbation to characterize RNA binding

The observed chemical shift changes for the RGG-box—TERRA24 titrations were calculated to quantify the binding affinity. The following formula was used, ${{{\rm{\delta }}_{{\rm{obs}}}} = \sqrt {{{( {\Delta {{\rm{\delta }}_{\rm{H}}}} )}^2} + {{( {\Delta {{\rm{\delta }}_{\rm{N}}}/5} )}^2}}}$, where $\Delta {{\rm{\delta }}_{\rm{H}}}$ and $\Delta {{\rm{\delta }}_{\rm{N}}}$ are the chemical shift changes in the ^1^H and ^15^N dimensions respectively. The apparent *K*_d_ for each residue was calculated using the observed chemical shift changes, as a function of the RNA concentration added to the protein sample at each titration step, using the following formula (implemented in Origin 9.0),

$$\begin{eqnarray*} \Delta \delta _{\rm{obs}} &=& \Delta \rm{\delta}_{\rm{max}}\Bigg([\rm{RGG]}_{\rm{T}} + [\rm{RNA}] + K_{\rm{d}} \\[5pt] &&- \sqrt{([\rm{RGG}]_{\rm{T}} + [\rm{RNA}] + K{\rm{_d})^2} - {4 [\rm{RGG}]_{\rm{T}}[\rm{RNA}]}} \Bigg)/2[\rm{RGG]_{\rm{T}}} \end{eqnarray*}$$

where $\Delta {{\rm{\delta }}_{{\rm{obs}}}}$ is the observed chemical shift change at a particular concentration of RNA added, $\Delta {{\rm{\delta }}_{{\rm{max}}}}$ is the total chemical shift change at free and fully saturated states, ${[ {{\rm{RGG}}} ]_{\rm{T}}}$ is the total protein concentration used for the titration experiment and $[ {{\rm{RNA}}} ]$ is the concentration of the RNA added at each step in the titration. The data was analyzed and the graphs were plotted using Origin 9.0.

## RESULTS

### UP1 and UP1+RGG interact with both single stranded RNA and TERRA RNA G-quadruplex

Supplementary Table S1 shows the RNA sequences used in this study. Although these RNA sequences have been studied previously (28,43), we deemed it necessary to characterize them under the current experimental conditions. Two RNA sequences: ssRNA-18 [GAGUAACCCGUAUCGUGA, 18mer] and a mutated TERRA sequence where two Gs are substituted with Cs in each repeat, ssTERRA-mut [(UUACCG)_4_, 24mer] were used as single stranded RNA control (Supplementary Table S1 and Figure 1C). TERRA-6 [UUAGGG, 6mer], TERRA-12 [(UUAGGG)_2_, 12mer] and TERRA-24 [(UUAGGG)_4_, 24mer] RNA sequences are designed to form tetrameric, dimeric, and intramolecular monomeric RNA G-quadruplexes of parallel topologies respectively (Supplementary Table S1 and Figure 1D-F). An abasic loop containing TERRA-24 sequence (abasicloopsTERRA-24), which was devoid of bases in the loop nucleotides of the RNA G-quadruplex, was also used in this study (Supplementary Table S1 and Figure 1G). Formation of G-quadruplex structures by these RNA sequences was confirmed by recording their CD and 1D ^1^H NMR spectra (for details, please see SI text, and Supplementary Figures S1A–C, S2A and B).

**Figure 2.**
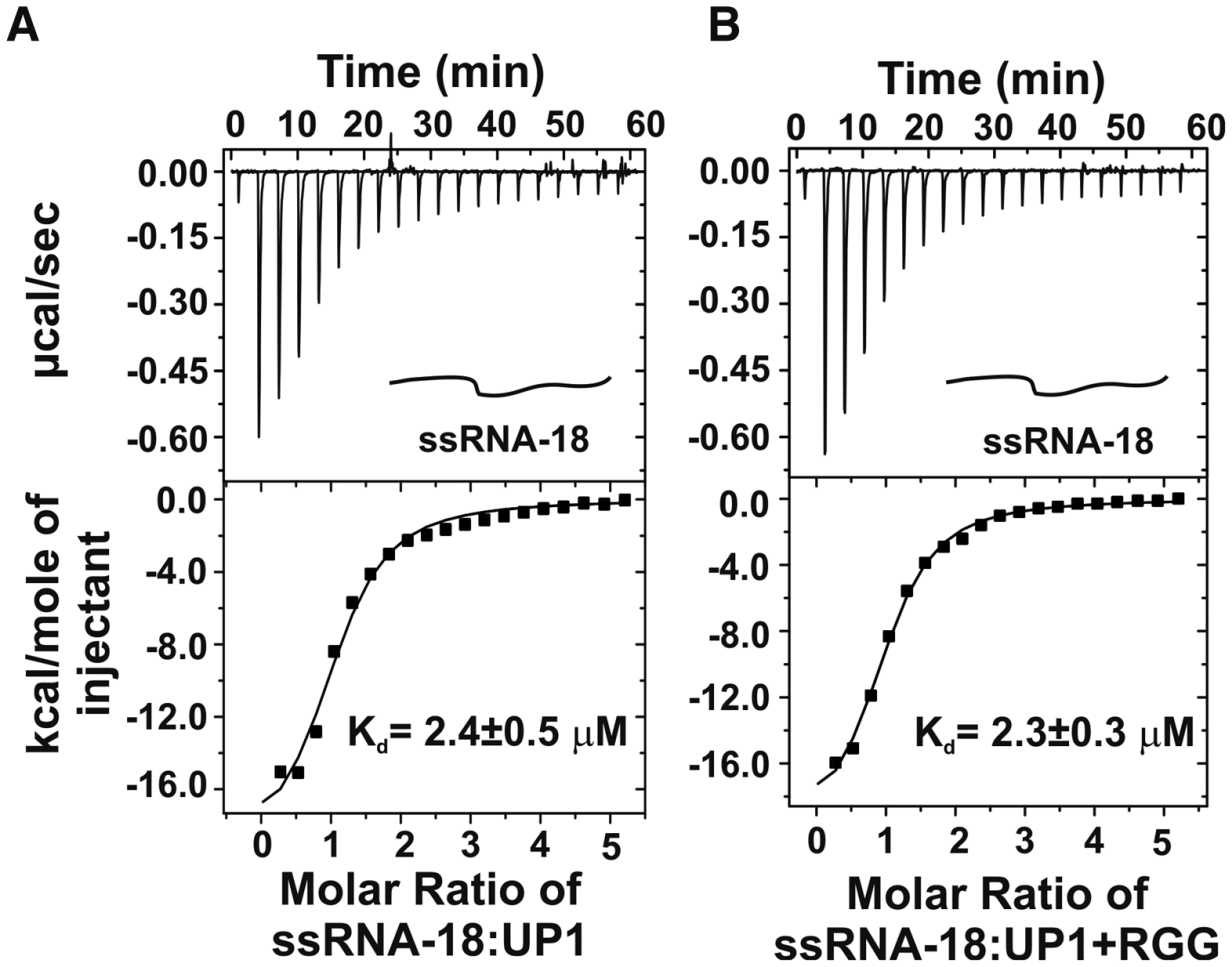
Raw and fitted ITC binding isotherms for the interaction of UP1 and UP1+RGG with single stranded RNA (ssRNA-18). (**A**) Interaction of single stranded ssRNA-18 RNA with UP1. (**B**) Interaction of single stranded ssRNA-18 RNA with UP1+RGG. The equilibrium K_d_s obtained upon fitting of the raw data is mentioned in each panel. Cartoon representations of the RNA used are shown in *inset*.

**Figure 3.**
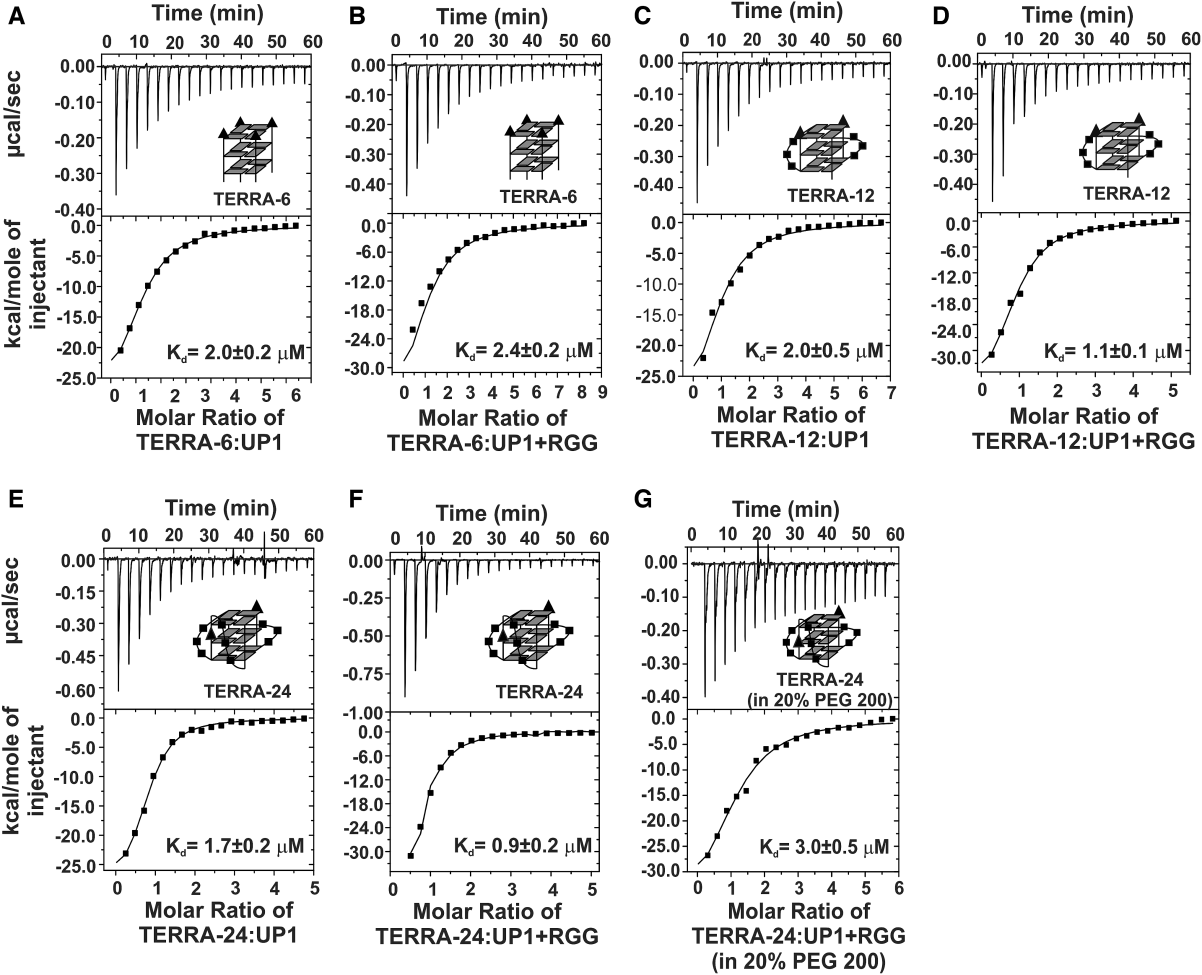
Raw and fitted ITC binding isotherms for the interaction of UP1 and UP1+RGG with different forms of TERRA RNA G-quadruplexes. (**A, B**) Interaction of TERRA-6 G-quadruplex with UP1 and UP1+RGG respectively. (**C, D**) Interaction of TERRA-12 G-quadruplex with UP1 and UP1+RGG respectively. (**E, F**) Interaction of TERRA-24 G-quadruplex with UP1 and UP1+RGG respectively. (**G**) Interaction of TERRA-24 RNA G-quadruplex with UP1+RGG in the presence of 20% PEG 200. The equilibrium K_d_s obtained upon fitting of the raw data is mentioned in each panel. Cartoon representations of the RNA G-quadruplexes used are shown in *inset*.

To delineate the roles of the UP1 domain and the RGG-box in TERRA RNA recognition (single strand and G-quadruplex form) we have used isothermal titration calorimetry (ITC) method. ITC directly measures the binding enthalpy upon complex formation and provides the information on equilibrium constant (*K*_d_), enthalpy change (Δ*H*), entropy change (Δ*S*), and stoichiometry (*n*) of the complex formation under the experimental conditions. For the ITC titrations, 5–10 μM of RNA solutions were titrated with 100–200 μM of UP1 (residues 1–196) and UP1 along with RGG-box (residues 1–249, henceforth called as UP1+RGG). All the interactions were exothermic at 25°C. Figures 2 and 3 show the raw and fitted ITC isotherms and Table 1 summarizes the results obtained after data integration and fitting. We observed a clear interaction and complex formation between UP1 and single stranded RNA (ssRNA-18) with a *K*_d_ of 2.37±0.49 μM and a stoichiometry of ∼1 (Figure 2A and Table 1). Similarly, we observed a clear interaction between UP1+RGG and ssRNA-18 with a *K*_d_ of 2.28±0.26 μM and a stoichiometry of ∼1 (Figure 2B and Table 1). Taken together, these results showed that UP1 and UP1+RGG bind single stranded RNA with similar affinities indicating that the UP1 domain constitutes the main binding site and the RGG-box (in UP1+RGG) likely has no significant contribution for the single stranded RNA binding.

**Table 1.** Equilibrium dissociation constants (*K*_d_s) and other thermodynamic parameters derived for interactions between RNA (single stranded RNA and G-quadruplexes formed by TERRA RNA repeats) and proteins (UP1, UP1+RGG and the isolated RGG-box) using ITC experiments

S.No.	Experiment	*K*_d_ (μM)	Δ*G* (kcal/mol)	Δ*H* (kcal/mol)	*T*Δ*S* (kcal/mol)	*n*
1	UP1:ssRNA-18	2.37±0.49	−7.67±1.58	−19.7±1.23	−12.03±2.81	1.04±0.05
2	UP1+RGG:ssRNA-18	2.28±0.26	−7.69±0.86	−20.4±0.71	−12.71±1.57	0.97±0.03
3	UP1:TERRA-6	2.02±0.17	−7.76±0.64	−31.31±1.4	−23.55±2.04	1.22±0.04
4	UP1+RGG:TERRA-6	2.38±0.23	−7.66±0.75	−38.84±2.74	−31.18±3.48	1.14±0.07
5	UP1:TERRA-12	2.04±0.47	−7.75±1.77	−36.38±5.61	−28.63±7.38	0.93±0.12
6	UP1+RGG:TERRA-12	1.07±0.14	−8.13±1.08	−43.01±2.48	−34.88±3.56	0.88±0.04
7	UP1:TERRA-24	1.71±0.17	−7.86±0.76	−29.66±0.95	−37.52±1.70	0.80±0.02
8	UP1+RGG:TERRA-24	0.92±0.17	−8.23±1.53	−41.88±2.81	−33.65±4.34	0.82±0.04
9	UP1+RGG:TERRA-24 (with 20% PEG 200)	3.0±0.55	−7.53±1.40	−40.89±4.15	−33.36±5.55	1.14±0.09
10	RGG:TERRA-24	9.0±4.3	−6.88±3.31	−0.67±0.27	6.21±3.58	1.06±0.34

### The RGG-box synergistically enhances the binding of UP1+RGG to TERRA RNA G-quadruplexes with loops in the structure

The interaction of UP1 and UP1+RGG with TERRA RNA G-quadruplexes was followed using ITC. Initially TERRA-6, that forms tetrameric G-quadruplex devoid of loops in its structure, was titrated with UP1 and UP1+RGG. We observed enthalpically driven interaction of UP1 and UP1+RGG with TERRA-6 RNA G-quadruplex with *K*_d_s of 2.02±0.17 and 2.38±0.23 μM respectively and a stoichiometry of ∼ 1 in both the cases (Figure 3A and B and Table 1). Similar values of K_d_s suggests that the RGG-box in UP1+RGG does not contribute significantly towards the binding of hnRNPA1 to tetrameric TERRA-6 RNA G-quadruplex and the UP1 domain constitutes the main TERRA-6 RNA G-quadruplex binding domain.

Loops containing dimeric TERRA-12 and monomeric intramolecular TERRA-24 RNA G-quadruplexes, were separately titrated with UP1 and UP1+RGG and the interactions were followed using ITC in the next sets of experiments. Both UP1 and UP1+RGG showed interaction with dimeric TERRA-12 and intramolecular TERRA-24 RNA G-quadruplexes. UP1 interacted with TERRA-12 and TERRA-24 with *K*_d_s of 2.04±0.47 and 1.71±0.17 μM respectively (Figure 3C and E). UP1+RGG interacted with TERRA-12 and TERRA-24 with *K*_d_s of 1.07±0.14 and 0.92±0.17 μM respectively (Figure 3D, and F and Table 1). In all the cases, we observed a stoichiometry of ∼1 for protein and RNA G-quadruplex interactions. It is evident that the presence of the RGG-box led to a small but consistent increase in the affinity of hnRNPA1 for dimeric TERRA-12 and monomeric TERRA-24 RNA G-quadruplexes. A larger enthalpy change (Δ*H*) was observed for UP1 and UP1+RGG binding to all three G-quadruplexes compared to the ssRNA-18 (data shown in Table 1). In general, specific protein-nucleic acid complex formation is enthalpically driven (44), therefore a large enthalpy change indicates a more specific protein - G-quadruplex complex formation (Table 1).

These results showed that UP1 binds to single stranded RNA and TERRA RNA G-quadruplexes of different topologies with similar *K*_d_s (Table 1). On the other hand, UP1+RGG binds the single stranded RNA and tetrameric TERRA-6 with affinities similar to UP1, however it binds dimeric TERRA-12 and intramolecular TERRA-24 RNA G-quadruplexes with consistently >2.4-fold better binding (Table 1). Taken together, our results show that both UP1 and UP1+RGG of hnRNPA1 interact with both single-stranded as well as RNA G-quadruplexes of different topologies, with the RGG-box region imparting an additive effect to the binding of the UP1 domain in case of the loop containing RNA G-quadruplexes.

In the next experiment, we titrated intramolecular TERRA-24 RNA G-quadruplex with UP1+RGG in the presence of 20% PEG 200, which is a crowding and dehydrating agent (Figure 3G). Small molecular weight PEG (e.g. PEG 200) has been shown to decrease the water activity, thereby facilitating the removal of the water molecules around the loops of the G-quadruplex (45). The formation of parallel G-quadruplex structure by TERRA-24 in the presence of 20% PEG 200 was confirmed by recording a CD spectrum of the RNA (SI text and Supplementary Figure S2A). We observed an enthalpically driven (Δ*H* = –40.89±4.15 kcal/mol) interaction between UP1+RGG and TERRA-24 in the presence of 20% PEG 200 with a *K*_d_ of 3.0±0.55 μM (Figure 3G and Table 1). Therefore, in the presence of 20% PEG 200 there is a reduction in binding affinity of UP1+RGG for TERRA-24 by ∼3 times. However, in comparison with TERRA-6, the binding of UP1+RGG for TERRA-24 in the presence of 20% PEG 200 did not drop significantly (similar *K*_d_s with the reported errors) (Table 1). These results point to the observation that likely the hydrated loop nucleotides in TERRA-24 contribute towards binding of RGG-box in hnRNPA1.

### The isolated RGG-box does not interact with the single stranded RNAs

Recently, we had shown that the isolated RGG-box of hnRNPA1 is an intrinsically disordered region of the protein (30). Poor chemical shift dispersion and repeated nature of the RGG sequences made it difficult to assign all the cross peaks in the 2D ^1^H–^15^N HSQC NMR spectra, but nonetheless the partially assigned spectra could be used to understand the interaction of the protein with RNA. To probe the specificity of the RGG-box towards single stranded and different forms of the TERRA RNA G-quadruplex, we used NMR chemical shift perturbation experiments. In a typical experiment, RNA solution was added in increasing amount to the uniformly ^15^N labeled protein (isolated RGG-box) and 2D ^1^H–^15^N HSQC spectrum was recorded at each step of the titration.

**Figure 4.**
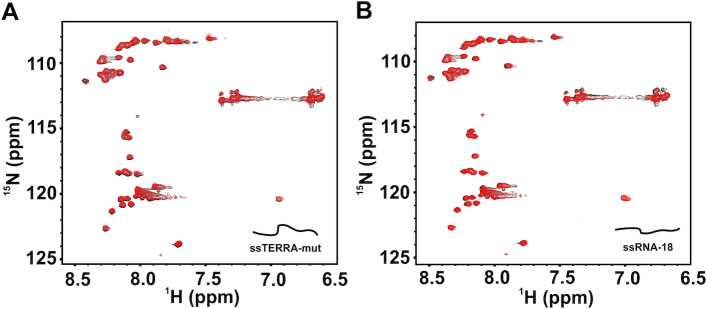
Interaction of the single stranded RNA sequences with the RGG-box of hnRNPA1 monitored using NMR spectroscopy. (**A**) 2D ^1^H–^15^N HSQC spectra of the free RGG-box (black) overlaid on to the spectra of RGG-box and ssTERRA-mut RNA complex at 1:2 (protein to RNA) molar ratio (red). (**B**) 2D ^1^H–^15^N HSQC spectra of the free RGG-box (black) overlaid on to the spectra of RGG-box and ssRNA-18 complex at 1:2 (protein to RNA) molar ratio (red).

We first titrated isolated RGG-box with increasing concentration of single stranded ssRNA-18 (up to 2 times molar excess) and recorded a 2D ^1^H–^15^N HSQC spectrum at each step of the titration. We observed no significant chemical shift perturbations in the 2D ^1^H–^15^N HSQC spectrum of the RGG-box (Figure 4A). In the next experiment, the RGG-box was titrated with the single stranded 24mer ssTERRA-mut RNA. Again we observed no significant chemical shift perturbations in the 2D ^1^H–^15^N HSQC spectrum of the RGG-box. NMR chemical shifts can report even very weak interactions (46). Therefore, lack of chemical shift perturbations observed here unambiguously showed that the isolated RGG-box does not bind to the single stranded RNA irrespective of its sequence (Figure 4A and B). This corroborates our ITC results presented earlier that showed no significant effect of the RGG-box on the binding of UP1+RGG (as compared to the UP1 alone) to the single stranded RNA (Figure 2A and B and Table 1).

### The isolated RGG-box exhibits structure specific interaction with the intramolecular TERRA RNA G-quadruplex

**Figure 5.**
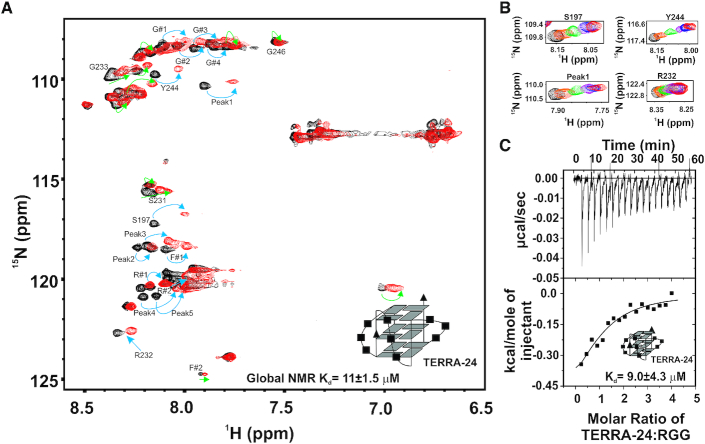
Interaction of the RGG-box of hnRNPA1 with the intramolecular TERRA-24 RNA G-quadruplex monitored using NMR spectroscopy. (**A**) 2D ^1^H–^15^N HSQC spectrum of the free protein (black) overlaid on to the spectrum of the RGG-box in complex with TERRA-24 at 1:3 (protein to RNA) molar ratio (red). The peaks undergoing perturbation in a fast-exchange timescale upon addition of RNA quadruplexes have been marked with cyan and green arrows. The peaks marked in cyan are common to Tel22 DNA G-quadruplex binding, reported in a previous study (30). The peaks marked in green represent the unique residues that are perturbed only in case of TERRA-24 RNA G-quadruplex binding. (**B**) Examples of few of the residues undergoing perturbation on adding TERRA-24 have been shown at protein to RNA molar ratios of 1:0 (black), 1:0.2 (orange), 1:0.5 (green), 1:1 (purple), 1:2 (blue) and 1:3 (red). The peaks shift continuously (unbound to bound complex) upon titrating the RNA G-quadruplex, which is a characteristic of fast exchange timescale kinetics. (**C**) Raw and fitted ITC binding isotherm for the interaction of the TERRA-24 RNA G-quadruplex with the RGG-box. The equilibrium *K*_d_ obtained upon fitting of the raw data is mentioned in the panel. Cartoon representations of the RNA used are shown in *inset* (A, C).

In the subsequent NMR titrations, we probed the binding of the isolated RGG-box to the TERRA RNA quadruplexes of different topologies. First, the ^15^N-uniformly labeled RGG-box was titrated with increasing concentration of physiologically relevant intramolecular TERRA-24 RNA G-quadruplex (43). We observed large chemical shift perturbations in the 2D ^1^H–^15^N HSQC spectrum of the RGG-box upon addition of TERRA-24 G-quadruplex RNA (Figure 5A). The RGG-box–TERRA-24 RNA G-quadruplex complex was in fast exchange time regime on NMR timescale, as we observed gradual shift in the positions of several resonance cross peaks upon addition of RNA (Figure 5B). This suggested a weak but specific interaction between the RGG-box and the intramolecular TERRA-24 G-quadruplex RNA. The perturbed cross peaks include identified glycine residues G#1-4, R#1-2, F#1, and the assigned residues Y244, S197 and R232. Some of these residues had been shown to interact with the telomere DNA G-quadruplex in a recent study (30). However, we observed additional residues of the RGG-box being perturbed upon binding to RNA G-quadruplex (Figure 5A, peaks marked with green arrows). Also, the magnitudes of the chemical shifts were greater for RNA G-quadruplex than the DNA G-quadruplex binding (please see Discussion section for more details) (Figure 5A).

The CSPs of the 14 residues were fit as a function of the RNA concentration added in each step of the titrations to calculate apparent dissociation constants for the individual fits (please see Materials and Methods and Supplementary Figure S3). The individual fit apparent *K*_d_s of these residues for TERRA-24 binding ranged from ∼3–18 μM. All individual fit *K*_d_s were fit to a global fit *K*_d_ of 11±1.5 μM.

The interaction between TERRA-24 and the RGG-box was also probed using ITC experiments at 25°C (Figure 5C and Table 1). We observed a clear complex formation between the isolated RGG-box and TERRA-24 G-quadruplex with a *K*_d_ of 9±4.3 μM, which is in agreement with the *K*_d_ value determined using NMR CPS fitting (*K*_d_ of 11±1.5 μM). Therefore, both ITC and NMR titration results unambiguously showed that the RGG-box of hnRNPA1 specifically interact with the intramolecular TERRA RNA G-quadruplex structures.

### RGG-box of hnRNPA1 interacts with the TERRA RNA G-quadruplex of different topologies

**Figure 6.**
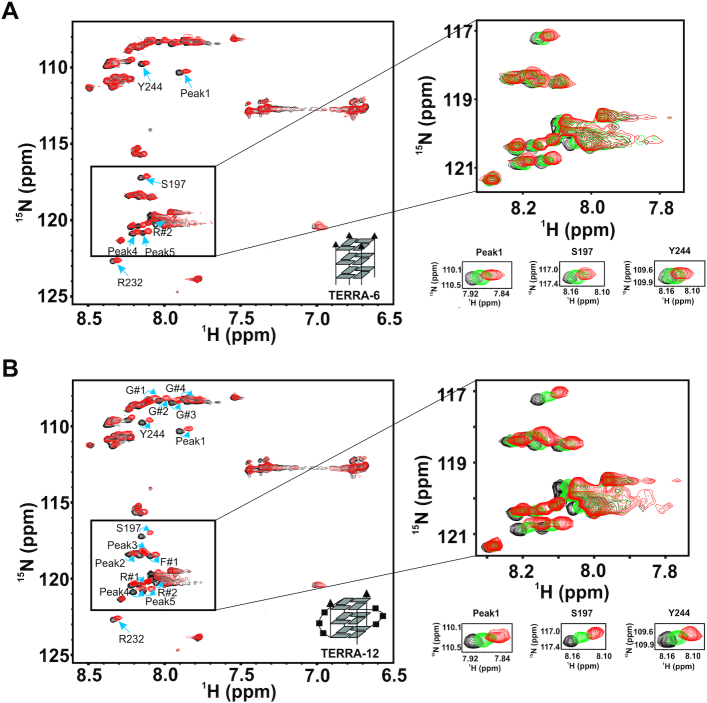
Interaction of the RGG-box of hnRNPA1 with tetrameric TERRA-6 and dimeric TERRA-12 RNA G-quadruplexes. (**A**) 2D ^1^H–^15^N HSQC spectrum of the free protein (black) overlaid on to the spectrum of the RGG-box in complex with tetrameric TERRA-6 RNA G-quadruplex at 1:2 (protein to RNA) molar ratio (red). Only a few peaks were seen to be perturbed with small chemical shift perturbations suggesting a very weak/transient interaction. A subset of the spectra, with peaks overlaid at protein to RNA molar ratios of 1:0 (black), 1:0.5 (green) and 1:2 (red) are shown in the *insets*. (**B**) 2D ^1^H–^15^N HSQC spectrum of the free protein (black) overlaid on to the spectrum of the RGG-box in complex with dimeric TERRA-12 RNA G-quadruplex at 1:1.5 (protein to RNA) molar ratio (red). A subset of the spectra with peaks overlaid at protein to RNA molar ratios of 1:0 (black), 1:0.5 (green) and 1:1.5 (red) are shown in the *insets*.

To gain further insights into the interaction of the RGG-box of hnRNPA1 with the RNA G-quadruplexes of different structure and topologies, we used tetrameric TERRA-6 that lacks loops in its structure and dimeric TERRA-12 G-quadruplex that has two loops in its topology (Figure 1D and E) in the next set of NMR titration experiments. TERRA-6 RNA G-quadruplex was titrated into a uniformly ^15^N labeled RGG-box and a 2D ^1^H–^15^N HSQC spectrum of the protein was recorded at each step. Interestingly, we observed very small chemical shift perturbations in few residues at an oversaturated 1:2 protein–TERRA-6 ratio (Figure 6A). This suggested that the tetrameric TERRA-6 G-quadruplex that lacks loops in the structure, interacts very weakly and transiently with the RGG-box of hnRNPA1. We did not observe any significant heat changes in the ITC experiments upon titration of TERRA-6 with the isolated RGG-box suggesting no binding and further augmenting the NMR titration results (Supplementary Figure S4). NMR chemical shifts are extremely sensitive to the environment and even very weak interactions can be observed using NMR. ITC on the other hand measures the binding enthalpy and therefore the interactions that proceed with very small heat changes (weak interactions) cannot be accurately measured using ITC.

In case of dimeric, loop containing TERRA-12 RNA G-quadruplex titrations, we observed distinct and significant chemical shift perturbations in several residues in the 2D ^1^H–^15^N HSQC spectrum of the RGG-box similar to RGG-box – TERRA-24 titration (Figure 6B). Taken together, these results showed specific binding of the RGG-box to the TERRA RNA G-quadruplexes (RGG-box interaction with TERRA-24 and TERRA-12 G-quadruplexes), where primarily the structured loops in the G-quadruplex structures likely constitute the primary determinants of binding. These observations support the ITC results, presented in the earlier section, which showed that the presence of the RGG-box in UP1+RGG results in its improved binding to TERRA-24 and TERRA-12 RNA G-quadruplexes (Table 1).

**Figure 7.**
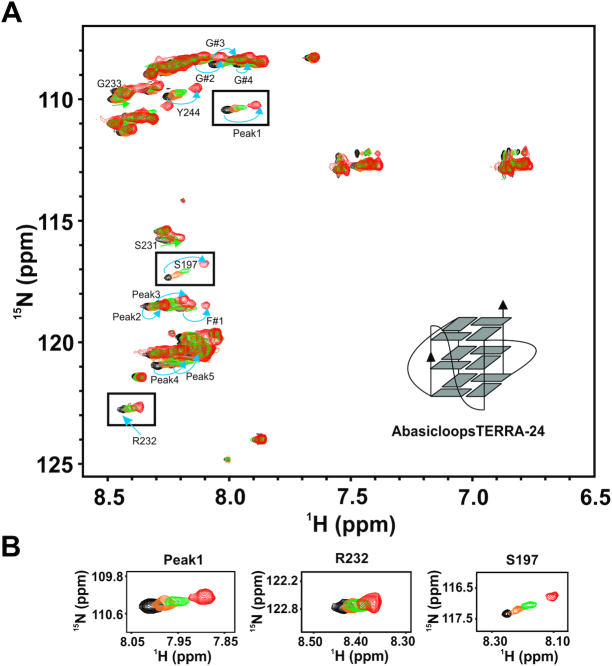
Interaction of the RGG-box of hnRNPA1 with intramolecular abasicloopsTERRA-24 RNA G-quadruplex. (**A**) 2D ^1^H–^15^N HSQC spectrum of the free RGG-box (black) and in complex with abasicloopsTERRA-24 RNA at 1:1 (protein to RNA) molar ratio (red). A cartoon representation of the intramolecular abasicloopsTERRA-24 G-quadruplex is shown. The specific chemical shift perturbations observed for several residues have been indicated with arrows. (**B**) Examples of a few residues of the RGG-box that are perturbed upon titrating with abasicloopsTERRA-24 have been shown. Overlay of peaks at four different protein to RNA molar ratios of 1:0 (black), 1:0.2 (orange), 1:0.5 (green) and 1:1 (red) are shown. The cross peak at ∼120.5 ppm in the ^15^N dimension and ∼7 ppm in the ^1^H dimension is not observed due to lower concentration of protein used in this experiments.

To further delineate the contribution of sugar-phosphate and nitrogen bases in the loop nucleotides of TERRA-24 G-quadruplex for the RGG-box binding, we designed a variant of TERRA-24 RNA that is devoid of bases in the loop nucleotides (called abasicloopsTERRA-24). AbasicloopsTERRA-24 contains only sugar phosphate backbone in the loop residues. The formation of G-quadruplex of parallel topology by abasicloopsTERRA-24 was validated through NMR and CD spectroscopy (Supplementary Figure S2B and C). The RGG-box was titrated with increasing concentration of abasicloopsTERRA-24 G-quadruplex and 2D ^1^H–^15^N HSQC spectrum was recorded at each step of the titration. A subset of the peaks, that were perturbed in case of titration with intramolecular TERRA-24 G-quadruplex, were perturbed in this titration (Figure 7A and B). The peaks that showed distinct chemical shift perturbation include R232, Peak 4, Peak 5, Peak 2, Peak 3, F#1, S197, S231, Peak1, Y244, G233, G#2, G#3 and G#4 (Figure 7A and B). Therefore, these results show that likely both the sugar-phosphate and the nitrogenous bases in TERRA-24 interact with the RGG-box.

### RGG-box enhances the RNA G-quadruplex unfolding ability of UP1

**Figure 8.**
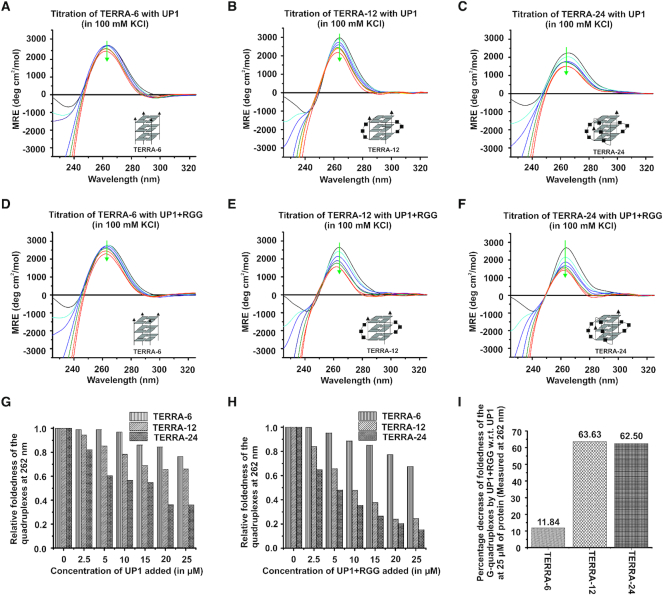
Unfolding of TERRA RNA G-quadruplex by UP1 and UP1+RGG monitored using CD spectroscopy. (**A–C**) Unfolding of tetrameric TERRA-6 (A), dimeric TERRA-12 (B), and intramolecular TERRA-24 (C) RNA G-quadruplexes by UP1. CD spectra of RNA G-quadruplexes are shown at 0 (black), 2.5 μM (cyan), 5 μM (purple), 10 μM (blue), 15 μM (green), 20 μM (orange) and 25 μM (red) of UP1. Cartoon representations of RNA used are shown in *inset*. The green arrow points the decrease in ellipticity at 262 nm, indicating the unfolding of G-quadruplex upon increasing concentration of the protein. (**D–F**) Unfolding of monomeric TERRA-6 (D), dimeric TERRA-12 (E), and intramolecular TERRA-24 (F) RNA G-quadruplexes by UP1+RGG. CD spectra of RNA G-quadruplexes are shown at 0 (black), 2.5 μM (cyan), 5 μM (purple), 10 μM (blue), 15 μM (green), 20 μM (orange) and 25 μM (red) of UP1+RGG. Cartoon representations of RNA used are shown in *inset*. The green arrow points the decrease in ellipticity at 262 nm, indicating the unfolding of G-quadruplex upon increasing concentration of protein. (G, H) Column graphs showing relative foldedness of TERRA-6, TERRA-12 and TERRA-24 with increasing concentration of UP1 (**G**) and UP1+RGG (**H**). (**I**) Column graph showing the percentage decrease in foldedness of TERRA-6, TERRA-12, and TERRA-24 RNA G-quadruplexes by UP1+RGG with respect to UP1 at 25 μM concentration of proteins.

UP1 is a well-established DNA G-quadruplex destabilizing protein (17,30,47). However, whether UP1 or UP1+RGG can destabilize TERRA RNA G-quadruplexes remained uncharacterized. We investigated the effect of UP1 and UP1+RGG on the destabilization/unfolding of TERRA RNA G-quadruplexes and monitored it using CD spectroscopy, a method that has been used extensively in understanding the structure and folding/unfolding of G-quadruplexes (48). Titration of 5 μM tetrameric TERRA-6, dimeric TERRA-12, and monomeric TERRA-24 RNA G-quadruplexes formed in 100 mM KCl, was carried out with increasing concentration of UP1 and UP1+RGG separately and a CD spectrum was recorded at each step of titration. A decrease in ellipticity at 262 nm (characteristic maxima for RNA G-quadruplex) in the CD spectra of RNA G-quadruplexes with increasing concentration of protein would indicate unfolding of the G-quadruplex structure. In case of TERRA-6 RNA G-quadruplex that lacks loops, we observed small structural changes in the CD spectra of TERRA-6 upon addition of UP1 (Figure 8A). On the other hand, in case of dimeric TERRA-12 and monomeric intramolecular TERRA-24 G-quadruplexes, we observed large and significant structural changes in the CD spectra upon addition of UP1 (Figure 8B and C). These results showed that UP1 unfolds the intramolecular TERRA-24 RNA G-quadruplex to a larger extent compared to the dimeric TERRA-12 RNA G-quadruplex, whereas the tetrameric TERRA-6 RNA G-quadruplex is unfolded to the least extent by UP1 (Figure 8A–C). The extent of RNA G-quadruplex unfolding by UP1 was compared by normalizing the CD signal at 262 nm and plotting it against the added UP1 concentration. This analysis showed that UP1 unfolds G-quadruplexes with loops (TERRA-12 and TERRA-24) with better efficiency than the RNA G-quadruplex that is devoid of loops (TERRA-6) (Figure 8G).

In the next set of experiments, we used UP1+RGG and monitored the unfolding of TERRA-6, TERRA-12 and TERRA-24 G-quadruplexes by recording CD spectrum at each step of the titration. Same trend in the unfolding of the RNA G-quadruplexes was observed: TERRA-24 was unfolded to the maximum extent followed by TERRA-12 and finally TERRA-6 at 1:5 molar ratio of RNA to protein (Figure 8D–F and H). The percentage decrease in foldedness of G-quadruplex structure by UP1+RGG with respect to UP1 was calculated at 25 μM of protein concentration. This analysis showed that UP1+RGG unfolds the loop containing RNA G-quadruplexes with better efficiency (with percentage decrease in foldedness of ∼65% for both TERRA-12 and TERRA-24 G-quadruplexes) than the UP1 domain alone (Figure 8I). The tetrameric TERRA-6 G-quadruplex structure was resistant to unfolding by both UP1 and UP1+RGG (as we observed only ∼ 12% decrease in foldedness by UP1+RGG compared to UP1 alone) (Figure 8A, D, I).

**Figure 9.**
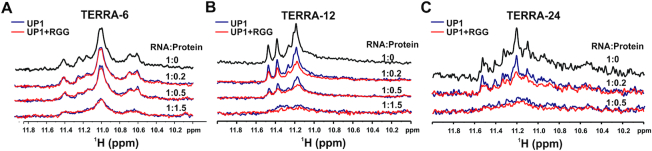
Unfolding of the different forms of the TERRA RNA G-quadruplex structures monitored through NMR spectroscopy. (**A**) Imino proton region of 1D ^1^H NMR spectra of tetrameric TERRA-6 G-quadruplex (in black) showing gradual loss of the imino proton peaks upon titration with UP1 (blue) and UP1+RGG (red). Incomplete disappearance of imino proton peaks was observed up to 1:1.5 (RNA to protein) molar ratio. (**B**) Imino proton region of 1D ^1^H NMR spectra of dimeric TERRA-12 G-quadruplex (black) showing gradual loss of the imino proton peaks upon titration with UP1 (blue) and UP1+RGG (red). Near complete disappearance of imino proton peaks was observed up to 1:1.5 (RNA to protein) molar ratio. (**C**) Imino proton region of 1D ^1^H NMR spectra of intramolecular TERRA-24 G-quadruplex (black) showing gradual loss of the imino proton peaks upon titration with UP1 (blue) and UP1+RGG (red). Near complete disappearance of imino proton peaks was observed up to 1:0.5 (RNA to protein) molar ratio.

We also monitored the unfolding of TERRA RNA G-quadruplexes by UP1 and UP1+RGG by NMR spectroscopy. The imino protons (resulting from the Hoogsteen H-bonded Gs in the G-quartet) in the 1D ^1^H spectra of different G-quadruplexes were followed at different ratio of RNA to proteins (UP1 or UP1+RGG). Disappearance of H-bonded imino peaks, therefore suggests unfolding of G-quadruplexes. In case of tetrameric TERRA-6 G-quadruplex, both UP1 and UP1+RGG destabilized the G-quadruplex structure, however inefficiently. The observation of imino proton peaks at 1:1.5 RNA to protein molar ratio suggest that both UP1 and UP1+RGG could not unfold TERRA-6 completely (Figure 9A). However, in case of loop containing dimeric TERRA-12, we observed that at 1:0.5 RNA to protein molar ratio UP1+RGG destabilized TERRA-12 to a greater extent than UP1, whereas at 1:1.5 RNA to protein molar ratio both the proteins unfold the TERRA-12 G-quadruplex (Figure 9B). Similarly, in case of loop containing intramolecular TERRA-24 G-quadruplexes, we observed that at 1:0.2 RNA to protein molar ratio, UP1+RGG destabilized TERRA-12 to a greater extent than UP1, whereas at 1:0.5 RNA to protein molar ratio both the proteins unfold the TERRA-12 G-quadruplex (Figure 9C).

We observed that the efficient unfolding of the TERRA RNA G-quadruplexes was achieved at higher RNA to protein ratio (lower protein concentrations) in NMR experiments compared to the CD titrations. The imino proton peaks monitored in the 1D ^1^H NMR spectra here emanate from the hydrogen bonded guanine residues in the G-quartets (49). During the unfolding of the G-quadruplex structure by hnRNPA1, the hydrogen bonds are likely to be disrupted before the disruption of the stacking interactions between the G-quartets. This may explain why we observed G-quadruplex unfolding at lower protein concentration in NMR as we observed initial disruption of Hoogsteen hydrogen bonded imino protons in 1D ^1^H NMR spectra.

Overall, the CD and NMR results showed that hnRNPA1 destabilizes the TERRA RNA G-quadruplexes, wherein the loop containing TERRA RNA G-quadruplexes (TERRA-24 and TERRA-12 are destabilized efficiently by the UP1 domain in the presence of the RGG-box. These observations are in sync with the ITC and NMR titration results that showed that the RGG-box imparts additional specificity to the UP1 towards its binding to loop containing TERRA RNA G-quadruplexes (Figures 3, 5, and 6). Therefore, we propose that the specific interaction of the RGG-box with TERRA RNA G-quadruplex helps the UP1 domain to destabilize the physiologically relevant intramolecular TERRA RNA G-quadruplex structures.

## DISCUSSION

hnRNPA1 is nucleic acids binding protein which binds to both DNA and RNA sequences, mainly by the N-terminal UP1 and the RGG-box domains (11). Both, telomere DNA as well as telomere repeats containing RNA (TERRA) are the binding substrates for hnRNPA1. G-rich telomere DNA and TERRA RNA sequences form higher order G-quadruplex structures readily in solution; however, the topologies as well as the stabilities of DNA and RNA G-quadruplexes in solution are distinct (50). While the telomere DNA forms G-quadruplexes of parallel, antiparallel or mixed topologies, the TERRA repeats usually form RNA G-quadruplex of parallel topology. Also, in general, the RNA G-quadruplexes are more stable structures compared to the DNA G-quadruplexes. This is attributed to the presence of 2′OH in the ribose sugar of RNA G-quadruplex that allows further intramolecular interactions and interactions with the water molecules (50,51). Indeed, we observed that the intramolecular TERRA-24 RNA G-quadruplex is thermally more stable (*T*_m_ = 80.5°C) than its equivalent intramolecular Tel22 DNA G-quadruplex (*T*_m_ = 68°C) under similar conditions (Supplementary Figure S1C) (30).

In case of protein and RNA G-quadruplex interactions, the 2′OH of the ribose sugar contributes towards additional hydrogen bond interactions (52). Further, it has been found that the contribution of sugar-phosphate in case of specific protein-RNA interactions is more pronounced than in the case of non-specific single stranded RNA – protein interactions (53). Therefore, RNA and DNA G-quadruplexes would act as distinct binding and unfolding substrates for hnRNPA1 and show unique thermodynamic profile upon interaction (17,30).

In this study, we observed that the UP1 domain of hnRNPA1 interacts with single-stranded RNA (18mer ssRNA-18 with *K*_d_ = 2.37±0.49 μM) with about two-fold lower affinity compared to the single-stranded DNA (22mer Tel22ss with *K*_d_ = 1.23±0.14 μM) under the same experimental conditions reported earlier (30). A similar trend in the binding was observed for UP1 in the presence of the RGG-box (UP1+RGG). However, in case of structured RNA G-quadruplexes, UP1 binds to the intramolecular TERRA-24 RNA G-quadruplex (*K*_d_ = 1.71±0.17 μM) with ∼3.3-fold higher affinity compared to the intramolecular Tel22 DNA G-quadruplex (*K*_d_ = 5.62±1.09 μM) reported earlier (30) under identical condition (in the presence of 100 mM KCl). The UP1 domain in the presence of the RGG-box (i.e. the UP1+RGG) showed ∼5.3-fold increased affinity towards intramolecular TERRA-24 RNA G-quadruplex (*K*_d_ = 0.92±0.17 μM) compared to intramolecular Tel22 DNA G-quadruplex (*K*_d_ = 4.85±0.60 μM) (30). A stoichiometry of ∼1 for UP1 (and UP1+RGG) and RNA (single stranded and G-quadruplex forms) association compared to a stoichiometry of ∼2 for UP1 and telomere DNA (single strand and G-quadruplex) association was observed (30). While, we speculate that the observed stoichiometry of hnRNPA1 for G-quadruplex stems from a complex interaction, the binding stoichiometry of UP1 for single stranded DNA and RNA sequences have been established using high resolution X-ray crystallography and NMR structural methods (54,55). Based on the existing structure of UP1 bound to single stranded telomere DNA repeats, we can visualize 2 molecules of UP1 binding to 1 molecule of about four TTAGGG repeats containing 22 nucleotides long DNA sequence accounting for 1:2 stoichiometry (30,54). However, a different mode of binding has been proposed for UP1 binding to RNA. For example, in a recent study, both RRM1 and RRM2 domains in UP1 were shown to simultaneously bind to a bipartite 21-nucleotide long RNA motif from human intronic splicing silencer ISS-N1 generating a 1:1 UP1–RNA complex (55). This may explain the observed binding stoichiometry of 1 between UP1 and 18 nucleotide long ssRNA-18 sequence used in this study using ITC experiments (Supplementary Figure S5).

In a recent study, using electrophoretic gel mobility shift assays (EMSA) and photo cross-linking experiments the interaction of hnRNPA1 with the single stranded RNA and TERRA G-quadruplexes was probed (28,29). These studies reported that while hnRNPA1 interacts with the RNA G-quadruplexes containing loops it shows no interaction with the single-stranded RNA or the TERRA-6 RNA G-quadruplex that is devoid of any loop. However, the precise role of the individual domains of hnRNPA1 for TERRA RNA (single stranded or G-quadruplex form) binding remained ambiguous. Here using robust ITC method, we have unambiguously shown that UP1 and UP1+RGG domains interact with both single-stranded RNA and TERRA-6 RNA G-quadruplex. Different experimental conditions and methods employed (photo cross linking/EMSA versus ITC) probably explain these differences. Furthermore, using NMR spectroscopy and ITC, here we showed that the RGG-box in hnRNPA1 is the main determinant that discriminates the binding between single stranded and G-quadruplex RNA.

**Figure 10.**
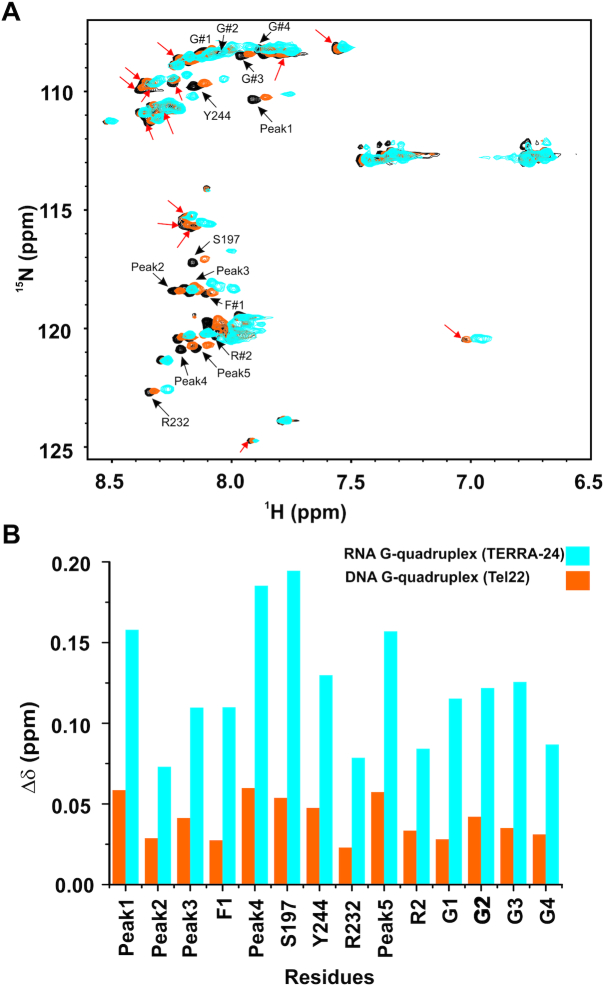
Comparison of the chemical shift perturbations in the residues of RGG-box of hnRNPA1 upon titration of intramolecular TERRA-24 RNA and intramolecular Tel22 DNA G-quadruplexes reported earlier (30). (**A**) Overlay of the free RGG-box of hnRNPA1 (black), RGG-box in complex with the TERRA-24 RNA G-quadruplex (cyan, at 1:3 molar ratio), and RGG-box in complex with the Tel22 DNA G-quadruplex (orange, at 1:6 molar ratio). The peaks undergoing perturbations specific to the TERRA-24 RNA G-quadruplex are shown by red arrows whereas the peaks, which are perturbed by both Tel22 DNA G-quadruplex and TERRA-24 RNA G-quadruplex are indicated by black arrows. (**B**) Comparative analysis of the chemical shift changes of the 14 residues of the RGG-box, which are common to Tel22 DNA and TERRA-24 RNA G-quadruplexes, at saturating protein to DNA or RNA concentrations.

The RGG-box is the second most frequently occurring RBDs in human genome after the RNA Recognition Motif (RRM domain) (56). Often, the RGG-motifs have been shown to enhance the binding affinity and activity of the adjacent domain for the nucleic acid binding and/or unfolding (56,57). The RGG-box—nucleic acid interactions are complex in nature, where the Arg and Gly residues in the RGG motifs, the number of amino acids interspersed between two RGG-repeats, and the Tyr/Phe residues adjacent to the RGG-motif seem to play important role (14,31,58–60). The RGG-box of hnRNPA1 is intrinsically disordered, which helps in DNA and RNA binding as well as promotes liquid–liquid phase separation (31). The isolated RGG-box of hnRNPA1 shows structure specific and loop dependent binding to both DNA (30) and RNA G-quadruplexes (results presented in this study). Interestingly, we observed more number of residues being perturbed and larger magnitude of perturbed CSPs in the 2D ^1^H–^15^N HSQC spectra of the RGG-box upon binding to TERRA-24 RNA G-quadruplex compared to Tel22 DNA G-quadruplex binding (Figure 10A and B). Based on the NMR CSP titration experiments, we deduced an apparent global fit *K*_d_ of 11±1.5 μM for the isolated RGG-box and TERRA-24 RNA G-quadruplex interaction, which is ∼ 31 times lower (stronger binding) than the isolated RGG-box interaction with Tel22 DNA G-quadruplex (*K*_d_ = 349 ± 35 μM) reported earlier (30). Therefore, the effect of the RGG-box on TERRA RNA G-quadruplex binding is greater than telomere DNA G-quadruplex binding.

Recently, it was proposed that the amide groups present in the backbone of glycine and the side chain of arginine residues of the RGG-box in different proteins are important for mediating protein and nucleic acid interaction (53). In addition, the aromatic amino acids that are often found adjacent to the core RGG-box also play a significant role in binding to the RNA bases through hydrophobic stacking, thereby playing role in mediating RNA-protein interactions (53). In this study we observed that all identified aromatic residues of the RGG-box are perturbed upon titration with the RNA G-quadruplex formed by TERRA-24. This includes two identified phenylalanine and two tyrosine residues. In addition, we showed that UP1 and UP1+RGG destabilize TERRA RNA G-quadruplex structures. Furthermore, the RNA G-quadruplex destabilization seems to be structure dependent since UP1 and UP1+RGG unfold loops containing TERRA-12 and TERRA-24 RNA G-quadruplex structures more efficiently compared to the tetrameric TERRA-6 G-quadruplex that is devoid of loops in its structure.

In summary, in this study we have reported the structure dependent recognition of TERRA RNA G-quadruplex by the RGG-box of hnRNPA1. We showed that the RGG-box of hnRNPA1 binds to the loops containing TERRA RNA G-quadruplexes but not with the single-stranded RNA. This provides the specificity to the UP1 domain to bind and enhances unfolding of the intramolecular TERRA RNA G-quadruplex. *In vivo*, the arginine residues in the RGG-box are known to undergo methylation (14,61–63). Therefore, arginine methylation is likely to influence the nucleic acid binding function of the RGG-box of hnRNPA1 in vivo. Our study provides new insights into the interplay of hnRNPA1, TERRA lncRNA, and telomere DNA that have implications in controlling the telomerase activity and telomere DNA maintenance.

## Supplementary Material

gkaa134_Supplemental_File
