# Supplementary material for: Structure specific recognition of telomeric repeats containing RNA by the RGG-box of hnRNPA1
Source: Nucleic Acids Res. 2020 Mar 4;48(8):4492–506. doi: 10.1093/nar/gkaa134 (PMC7192615; doi:10.1093/nar/gkaa134)
Supplement: gkaa134_Supplemental_File [file gkaa134_supplemental_file.pdf]

Supplementary Information for:

Structure specific recognition of telomeric repeats containing RNA by the RGG-box of hnRNPA1

Meenakshi Ghosh[1] and Mahavir Singh[1,2,*]

[1]Molecular Biophysics Unit, Indian Institute of Science, Bengaluru, 560012, INDIA

[2]NMR Research Centre, Indian Institute of Science, Bengaluru, 560012, INDIA

* To whom correspondence should be addressed

Email: singh@iisc.ac.in

This PDF includes:

Supplementary text

Figures S1-S5

Table S1

References for SI

Supplementary text

Characterization of TERRA RNA G-quadruplexes

Table S1 shows the RNA sequences that were used in this study. Although these RNA sequences have been studied previously (1, 2), we deemed it necessary to characterize them under the current experimental conditions. Two RNA sequences were used as single stranded RNA control: an 18mer control RNA sequence (ssRNA-18) and a 24mer single stranded mutated TERRA sequence where two Gs are substituted with Cs (ssTERRA-mut) (Table S1). These two RNA sequences are designed so that they do not form G-quadruplex and remain in the single stranded form in solution. 6mer TERRA-6, 12mer TERRA-12, and 24mer TERRA-24 RNA sequences are designed to form tetrameric, dimeric, and monomeric intramolecular RNA G-quadruplexes of parallel topologies respectively (Table S1). An abasic loop containing TERRA-24 sequence (abasicloopsTERRA-24) was devoid of bases in the loop residues of the RNA, was also used in this study (Table S1).

The CD spectra of the G-quadruplexes formed by the TERRA-6, TERRA-12 and TERRA-24 RNA sequences were acquired in physiologically relevant 100 mM KCl containing buffer (3). TERRA-6 sequence forms a tetrameric, parallel G-quadruplex without loops in its structure. TERRA-12 sequence adopts a dimeric, parallel G-quadruplex structure with two loops and TERRA-24 sequence adopts a monomeric, intramolecular, parallel G-quadruplex structure with three loops (1, 2). All the three RNA sequences form G-quadruplexes of parallel conformation that is characterized by positive maxima at 262 nm and minima at 240 nm in the CD spectra (Fig. S1A). Further, the presence of peaks in the imino region of 1D ^{1}H NMR spectra confirms the formation of Hoogsteen hydrogen bonded G-quadruplex structures by these RNA sequences (Fig. S1B). The CD spectra of ssRNA-18 and ssTERRA-mut sequences corresponded to that of single-stranded RNA. This was further confirmed by recording the 1D ^{1}H NMR spectra that showed absence of hydrogen bonded imino peaks in the spectra (Fig. S1B).

The thermodynamic stabilities of TERRA-6, TERRA-12, and TERRA-24 RNA G-quadruplexes were monitored using CD thermal melting experiments in the presence of 100 mM KCl. The CD signal at 262 nm was followed as a function of increasing temperature. In all the cases, we observed two state, sigmoidal melting transitions. The T_m of the intramolecular G-quadruplex, TERRA-24 was calculated to be 80.5°C (Fig. S1C). In a previous study, a T_m of 64.6°C in the presence of 10 mM KCl had been reported for this sequence (1, 2). This confirms the influence of potassium concentration on the thermal stability of the RNA G-quadruplex (1, 4). Under the

same condition, dimeric TERRA-12 G-quadruplex exhibited a T_m of 63.4°C, whereas the tetrameric TERRA-6 G-quadruplex exhibited a T_m of 72.5°C. These results show that in the presence of 100 mM KCl, intramolecular TERRA-24 forms thermally most stable G-quadruplex structure followed by TERRA-6 and TERRA-12 structures respectively.

The formation of the RNA G-quadruplex structure by TERRA-24 in the presence of 20% PEG 200 was confirmed using CD spectroscopy (Fig. S2A). Formation of an intramolecular, pralallel G-quadruplex by the abasicloopsTERRA-24 RNA sequence was confirmed by CD spectroscopy (Fig. S2B) and 1D ^{1}H NMR spectroscopy (Fig. S2C).

ITC titration of TERRA-6 RNA with UP1+RGG

The interaction of UP1+RGG with RNA G-quadruplex was investigated using ITC. However no significant heat change was observed that suggests no interaction between UP1+RGG and TERRA-6 as probed using ITC (Fig. S4).

Supplementary Figures

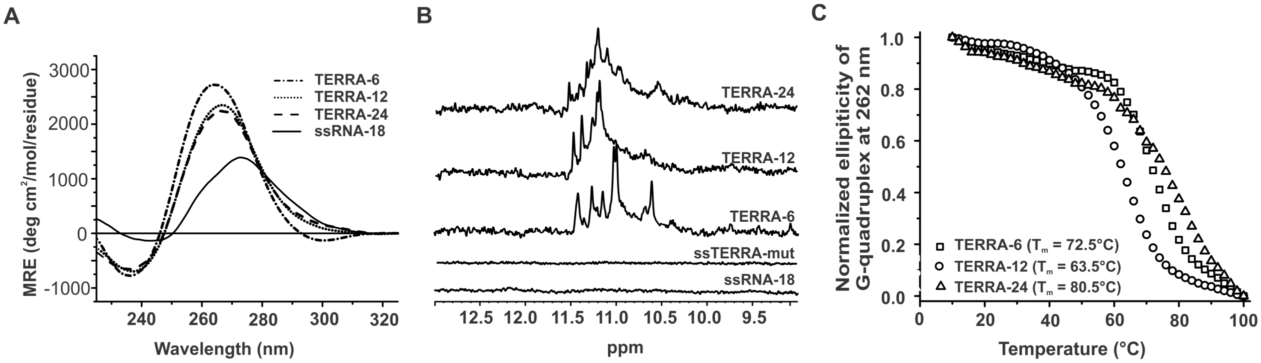

Figure S1. Characterization of TERRA RNA repeats. (A) CD spectra of different ssRNA-18, TERRA-6, TERRA-12, and TERRA-24 RNA sequences used in this study. (B) Imino region of 1D ^{1}H NMR spectra of the monomeric TERRA-6, dimeric TERRA-12, and intramolecular monomeric TERRA-24 RNA G-quadruplexes. (C) Thermal melting of TERRA-6, TERRA-12, and TERRA-24 RNA G-quadruplexes monitored by CD spectroscopy. The calculated thermal melting transition temperatures (T_m) are mentioned in the figure (C).

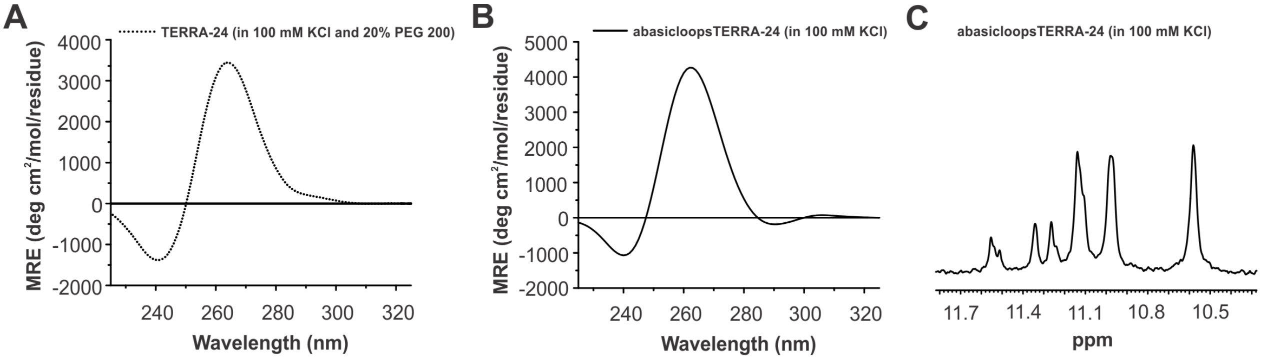

Figure S2. Characterization of RNA G-quadruplex formed by TERRA-24 in the presence of 20% PEG 200 and abasicloopsTERRA-24. (A) CD spectra of TERRA-24 in the presence of 20% PEG 200. (B) CD spectra of abasicloopsTERRA-24 RNA. (C) Imino region of 1D ^{1}H NMR spectrum of the abasicloopsTERRA-24 RNA G-quadruplex.

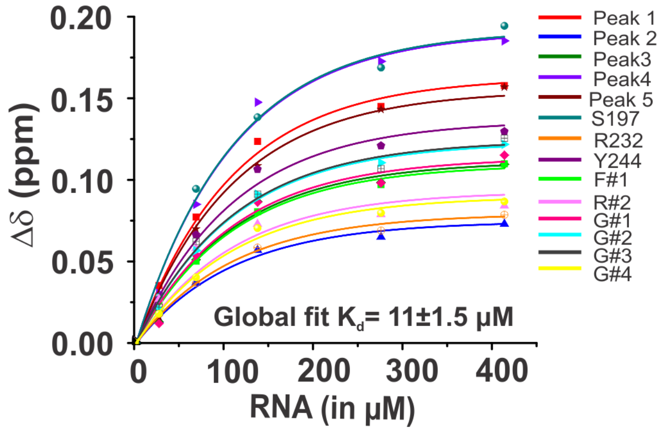

Figure S3. Interaction of the RGG-box of hnRNPA1 with the intramolecular TERRA-24 RNA G-quadruplex monitored using NMR spectroscopy. The chemical shift changes of the select residues plotted against the increasing concentration of TERRA-24 added to the RGG-box. The individual fit dissociations constants of the residues were fit to a global fit K_d of 11±1.5 μM.

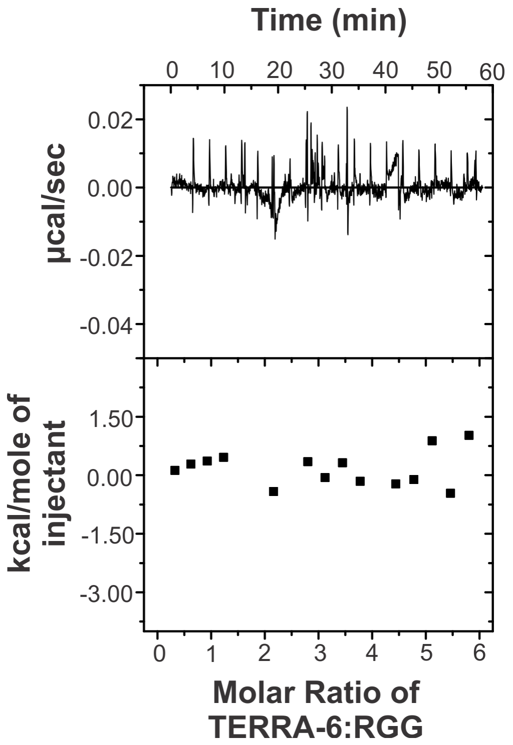

Figure S4. ITC isotherm for the titration of TERRA-6 G-quadruplex with the RGG-box of hnRNPA1. No appreciable heat change was observed during the titration.

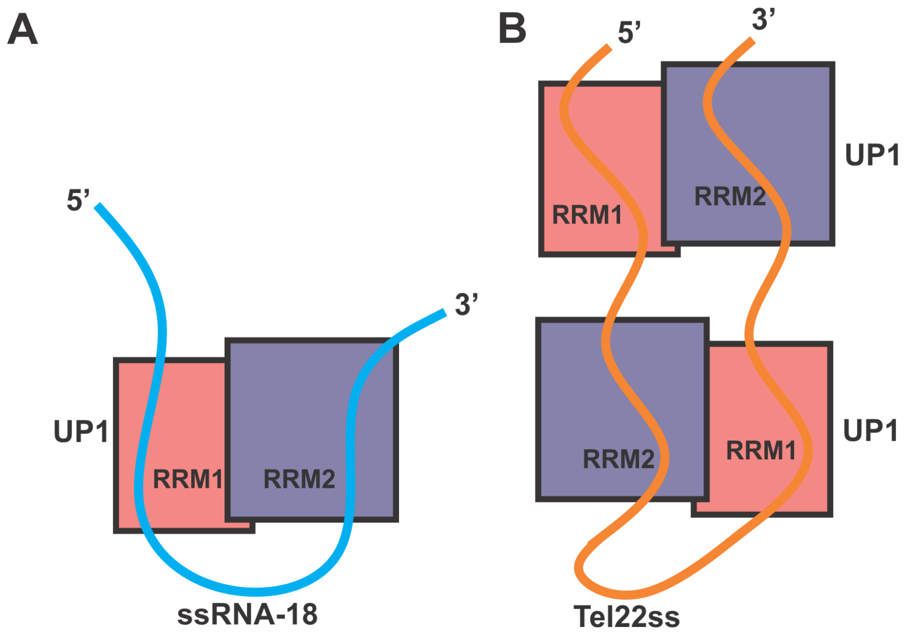

Figure S5. Plausible mode of interaction of UP1 domain (consisting of RRM1 and RRM2) with the single stranded RNA (18mer ssRNA-18 in blue) (A) and single stranded DNA (22mer Tel22ss in orange) (B).

Supplementary Table

Table S1. RNA sequences used in this study.

DNA	Sequence
ssRNA-18 (18 nt)	GAGUAACCCGUAUCGUGA
ssTERRA-mut (24 nt)	$(UUACCG)_4$
TERRA-6 (6 nt)	UUAGGG
TERRA-12 (12 nt)	$(UUAGGG)_2$
TERRA-24 (24 nt)	$(UUAGGG)_4$
Abasicloops TERRA-24 (24 nt)	[/rSpacer//rSpacer//rSpacer/-GGG]$_4$<br><br>where /rSpacer/ is a ribose sugar without any base.

Supplementary References

1. Biffi G, Tannahill D, & Balasubramanian S (2012) An intramolecular G-quadruplex structure is required for binding of telomeric repeat-containing RNA to the telomeric protein TRF2. *J Am Chem Soc* 134(29):11974-11976.
2. Liu X*, et al.* (2017) Structure-Dependent Binding of hnRNPA1 to Telomere RNA. *J Am Chem Soc* 139(22):7533-7539.
3. Su DG, Fang H, Gross ML, & Taylor JS (2009) Photocrosslinking of human telomeric G-quadruplex loops by anti cyclobutane thymine dimer formation. *Proc Natl Acad Sci U S A* 106(31):12861-12866.
4. Garavis M*, et al.* (2013) Mechanical unfolding of long human telomeric RNA (TERRA). *Chemical communications* 49(57):6397-6399.
